# Incidence and predictors of early posttraumatic seizures among patients with moderate or severe traumatic brain injury in Northwest Ethiopia: an institution-based prospective study

**DOI:** 10.1186/s12883-024-03536-z

**Published:** 2024-01-24

**Authors:** Nega Dagnew Baye, Fikadie Dagnew Baye, Assefa Agegnehu Teshome, Atalo Agimas Ayenew, Anmut Tilahun Mulu, Endeshaw Chekol Abebe, Zelalem Tilahun Muche

**Affiliations:** 1 Department of Biomedical Sciences, College of Health Sciences, Debre Tabor University, P.O. Box:272, Debretabor, Ethiopia; 2 Department of Pediatrics and Child Health, College of Health Sciences, Debre Tabor University, Debretabor, Ethiopia

**Keywords:** Traumatic brain injury, Seizures, Incidence, Ethiopia

## Abstract

**Background:**

Early posttraumatic seizure (PTS) is a well-known complication of traumatic brain injury (TBI) that can induce the development of secondary brain injuries, including increased intracranial pressure, brain death, and metabolic crisis which may result in worse outcomes. It is also a well-recognized risk factor for the development of late post-traumatic seizure and epilepsy. This study was aimed to assess the incidence and predictors of PTS among patients with moderate or severe TBI admitted to Debre Tabor Comprehensive Specialized Hospital, Northwest Ethiopia.

**Methods and setting:**

An institutional-based prospective follow-up study was conducted on 402 patients with TBI admitted to the neurologic unit from June 1, 2022 to January 30, 2023. A systematic sampling technique was employed. The incidence rate of occurrence of early PTS was calculated. Both bivariable and multivariable Cox proportional hazard regression was performed. The strength of the association was measured using adjusted hazard ratios with a 95% confidence interval and *p*-values < 0.05.

**Results:**

The incidence rate of early PTS was 2.7 per 100 person-days observation. Early PTS was observed in 17.7% of TBI patients. Age 75 and above (AHR = 2.85, 95%CI: 1.58–5.39), severe TBI (AHR = 2.06, 95%CI: 1.03–3.71), epidural hematoma (AHR = 2.4, 95% CI: 1.28–4.57), brain contusion (AHR = 2.6, 95%CI: 1.07–4.09), surgical intervention (AHR = 1.7, 95%CI: 1.03–3.82), posttraumatic amnesia (AHR = 1.99, 95%CI: 1.08–3.48), history of comorbidities (AHR = 1.56, 95%CI: 1.08–3.86), and history of alcohol abuse (AHR = 3.1, 95%CI: 1.89–5.23) were potential predictors of early PTS.

**Conclusion:**

The incidence of early PTS was high. Since, early PTS can worsen secondary brain damage, knowing the predictors helps to provide an effective management plan for patients likely to develop early PTS and improve their outcome.

## Background

Traumatic brain injury (TBI) is the most common cause of a neurosurgical emergency as well as the leading cause of disability and death in young adults globally, with a devastating impact on patients and their families [1]. Patients who survive the acute phase of TBI typically have a greater risk of developing disabilities and comorbidities later in life, and has a severe impact on their life span [2]. The most common complications associated with TBI include seizures, cognitive impairment, hydrocephalus, cerebrospinal fluid leakage, Parkinson’s disease, Alzheimer’s disease, dementia pugilistica, and posttraumatic epilepsy [3].

According to recent research, seizure was found to be an important contributor to premature death among individuals who were hospitalized and received inpatient rehabilitation for TBI [4]. The major complication after TBI is post-traumatic seizure (PTS). It can occur any time post-TBI and can be immediate PTS (occurring within 24 h), early PTS (occurring from first to 7 days), and late PTS (occurring after 7 days of injury) [5, 6].

Early PTS is a well-known complication of TBI that can induce the development of secondary brain injury, including increased intracranial pressure, brain atrophy, worsening cerebral edema, and impaired brain metabolism [7]. This could pose a significant challenge for the patient during the period of critical care and result in worse outcomes, including longer hospital stays and poorer functional outcomes in the longer-term [8–13]. In addition, early PTS appears to increase morbidity and mortality in the early stages following TBI [9, 14]. It is also a well-recognized risk factor for the development of late post-traumatic seizure with a higher relative risk of developing epilepsy than the general population [2, 11, 15].

Evidences shows that early PTS incidence varied significantly from 0.4 to 26.7% in different studies depending on the study population and methods for seizure detection [2, 7, 9, 11, 14, 16–20]. In Nigeria, the incidence of early PTS was 10.1% [21]. Also, a previous study of PTS in cohorts of Nigerian patients was 11.9% [22].

According to studies factors including age, TBI severity, medical comorbidities, subdural hemorrhage, epidural hematoma (EDH), brain contusion, and chronic alcohol abuse have been identified as predictors for early PTS [7, 9, 14, 16, 17].

In Ethiopia, evidence-based information about the incidence and predictors of posttraumatic seizure among patients with TBI remains scant. Therefore, this study aimed to assess the incidence and predictors of early PTS among patients with TBI.

## Materials and methods

### Study design and setting

A hospital-based prospective follow-study was conducted among 402 patients diagnosed with moderate or severe TBI hospitalized in Debre Tabor Comprehensive Specialized Hospital from June 1, 2022 to January 30, 2023. The hospital is found in Debre Tabor town which is located 667 km from Addis Ababa and 98 km from Bahir Dar and is the zonal center of South Gondar Zone, Northwest Ethiopia. The hospital serves over 2.2 million people from the surrounding area and provides a broad range of medical services for all age groups [23].

### Study subjects and eligibility criteria

Patients with moderate or severe TBI hospitalized in the neurologic ward of the Hospital were the study population.

All patients diagnosed with moderate or severe TBI who were aged 18 years and older and consented to participate in the study were included in the study. While, patients with a history of previous TBI, seizures, brain tumor, or stroke were excluded from this study.

### Variables

Occurrence of early PTS was the primary outcome. Demographics (age, sex, religion, educational status, marital status, and place of residence), clinical and injury-related characteristics (localization of injury, severity of TBI at admission, cause of TBI, brain neuroimaging findings, history of comorbidities, mode of intervention, posttraumatic amnesia), and behavioral characteristics like chronic alcohol use were the predictor variables. Educational status was labeled as: no formal education, primary education which entails 8 years of formal education, secondary education which entails 12 years of formal education, and college and above. The mode of neuroimaging (which allows to visualize the type and extent of brain damage after a TBI) was CT or MRI ordered by neurologists working in the hospital. Place of residence is labeled as a rural place of residence when participants are from low-density areas where households are often several kilometers apart heavily engaged in agriculture and typically lack access to electricity, piped water, or improved roads and urban place of residence when patients are from a high-density town with close access to a variety of amenities including university, teaching hospital, various clinics, schools, places of worship, electricity, and business sectors.

### Sample size determination and sampling technique

The required sample size was computed using a single population proportion formula by considering the incidence of early PTS as 50%, 95% confidence interval (CI), 5% margin of error, and a 10% non-response rate. The final sample size was 422. Based on the eligibility criteria, a systematic random sampling technique was employed until the necessary samples were obtained.

### Operational definitions

#### EPTS

is defined as at least one seizure observed or described by health care professionals occurring 24 h to 7 days after TBI [17].

#### Comorbid diseases

A patient with TBI who had preexisting hypertension and/or diabetes mellitus, which were assessed by reviewing the patient’s medical records and self-report.

#### Moderate TBI

Glasgow Coma Scale (GCS) 9 to 12 and loss of consciousness 30 min to 24 h [12, 24].

#### Severe TBI

GCS 3 to 8 and loss of consciousness for more than 24 h [12, 24].

### Data collection and quality assurance

Data were collected by using structured questionnaire, which was adapted and prepared after reviewing relevant literatures. The questionnaire was prepared first in English and translated into Amharic and then translated back to English to ensure the accuracy of the translated version. The questionnaires included baseline demographics, behavioral factors, clinical and injury-related characteristics. The data were collected by six medical doctors who work in the hospital. Three days of training were also given to data collectors. Data collectors follow participants daily for clinical seizures for the first 7 post-injury days. Daily supervision of data collectors was done during the data collection period.

### Study procedures

The follow-up for occurrence of early PTS started immediately after patients were diagnosed and admitted to the neurologic ward of the hospital. Immediately at admission, data collectors asked the patient/caregiver for written informed consent and then enrolled. The follow-up time of the patient ranged from the 1st to 7 days until the development of early PTS.

### Statistical analysis

STATA 14 statistical software was used to analyze the data. Descriptive statistics such as frequency, percentage, mean, and standard deviation were used to describe the characteristics of study participants, which were then summarised and presented in text and tables. The equality of survivor functions was assessed by a log-rank test and the incidence rate of early PTS was calculated. Bivariable Cox proportional hazard regression was performed. Then, variables with a *p*-value < 0.25 were included in a multivariable Cox proportional hazard regression analysis to find the predictors of early posttraumatic seizure. The assumption was met under Schoenfeld residuals proportional hazard assumption test for each variable and the overall global test (*p*-value > = 0.05). The strength of the association was measured using adjusted hazard ratios (AHR) with a 95% confidence interval and *p*-values of < 0.05.

## Results

### Patients’ characteristics

A total of 402 patients with moderate or severe TBI were enrolled in the study, with a response rate of 95.3%. The mean age of participants was 44.1 years (SD-13.7 years). One hundred eighty (44.8%) patients were in the age range of 40–59. Two hundred and fifty-five (63.4%) patients were males and 145 (36.1%) participants attended college or above (Table 1).

**Table 1:** Socio-demographic characteristics of patients with TBI

Variables		Frequency	Percentage
Age	18–39	157	39.1%
	40–59	180	44.8%
	60–79	56	13.9%
	80 and above	9	2.2%
Sex	Male	255	63.4%
	Female	147	36.6%
Marital status	Married	234	58.2%
	Single	108	26.9%
	Widowed	37	9.2%
	Divorced	23	5.7%
Level of education	No formal education	37	9.2%
	Primary education	101	25.1%
	Secondary education	119	29.6%
	College and above	145	36.1%
Religion	Orthodox	338	83.8%
	Protestant	27	6.7%
	Muslim	31	7.7%
	Other	7	1.8%
Place of residence	Rural	156	38.8%
	Urban	246	61.2%

### Clinical and injury-related characteristics

Of all patients with TBI, the majority of injury cases (189, 47%) were due to road traffic accidents followed by physical assault (146, 36.3%). About 165 (41%) patients had severe TBI and 34.3% were injured in the left lateral part of their brain. The majority of the patients, (298, 74.1%) had brain neuroimaging done and 276 (92.6%) of them had an abnormal finding. Among the findings, EDH was the most common (136, 49.3%) followed by brain contusion (41, 14.9%). Seventy-three (18.2%) patients had a history of preinjury secondary medical diagnosis, of which 44 patients had hypertension and 29 had diabetes mellitus (Table 2).

**Table 2:** Clinical and injury-related characteristics of patients with TBI

Variables		Frequency	Percentage
Cause of TBI	Road traffic accident	189	47%
	Assault	146	36.3%
	Falls	67	16.7%
GCS	Moderate (9–12)	237	59%
	Severe ( < = 8)	165	41%
Anti-seizure prophylaxis given	Yes	269	66.9%
	No	133	33.1%
Brain neuroimaging findings	Skull fracture	28	9.7%
	EDH	136	49.3%
	Subdural hematoma	37	13.4%
	Subarachnoid hemorrhage	35	12.7%
	Brain contusion	41	14.9%
Localization of injury	Frontal	91	22.7%
	Right lateral	124	30.8%
	Left lateral	138	34.3%
	Occipital	49	12.2%
Mode of intervention given	Surgical	104	25.9%
	Non-surgical	298	74.1%
Type of TBI	Penetrating	38	9.5%
	Blunt	345	85.8%
	Penetrating and blunt	19	4.7%
Posttraumatic amnesia	Yes	156	38.8%
	No	246	61.2%
History of comorbidities*	Yes	73	18.2%
	No	329	81.8%
Chronic alcohol use	Yes	107	26.6%
	No	295	73.4%

*Hypertension and diabetes mellitus

### Incidence of early PTS

During the follow-up, 71 (17.7%) patients with TBI develop early PTS. The median time to development of early PTS was 5 days (95% CI = 3.8, 6.1). Patients were followed for a minimum of 2 days to a maximum of 7 days, which provides a total of 2598 person-days of observation and a 0.0273 incidence rate. The Incidence rate of early PTS was 2.7 per 100 Person -days of observation. The majority of them had severe TBI (44, 62%).

### Predictors of early PTS

Variables with a *p*-value < 0.25 on bivariable Cox proportional hazard regression were entered into a multivariable Cox proportional hazard regression to control possible confounders and to identify significant predictors of early PTS. Accordingly, older age, severe TBI, epidural hematoma, brain contusion, surgical intervention, post-traumatic amnesia, history of comorbidities, and alcohol abuse were independently associated with early PTS in multivariable Cox proportional hazard regression.

The probability of developing early PTS was higher (AHR = 2.85, 95% CI: 1.58–5.39) among older age patients with TBI. The hazard of developing early PTS for those patients with TBI who are alcohol abusers was 3.1 times higher (AHR = 3.1, 95% CI: 1.89–5.23) when compared to those who are non-users. Patients who had severe TBI showed 1.88 times (AHR = 1.88, 95% CI: 1.07–4.02) higher risk of developing early PTS than patients with moderate TBI. Patients with TBI who had EDH showed (AHR = 3.33, 95% CI: 1.85–6.17) higher risk of developing early PTS (Table 3).

**Table 3:** Cox-proportional hazards regression analysis to determine the predictors of early PTS among patients with TBI

Variables	Early PTS		AHR (95% CI)	*P*-value
	Yes (%)	No (%)		
**Age**				
18–39	17 (10.8%)	140 (89.2%)	1	
40–59	21 (11.7%)	159 (88.3%)	1.06 (0.34–3.42)	0.06
60–79	26 (46.4%)	30 (53.6%)	2.04 (1.13–3.66)	0.003*
80 and above	7 (77.8%)	2 (22.2%)	2.85 (1.58–5.39)	0.00*
**Sex**				
Female	32 (21.8%)	115 (78.2%)	1	
Male	39 (15.3%)	216 (84.7%)	1.73 (0.69–3.57)	0.08
**GCS at presentation**				
Moderate	27 (11.3%)	210 (88.7%)	1	
Severe	44 (26.7%)	121 (73.3%)	2.06 (1.03–3.71)	0.04*
**Brain neuroimaging findings**				
Skull fracture	5 (17.9%)	23 (82.1%)	1	
EDH	39 (29%)	97 (71%)	2.4 (1.28–4.57)	0.00*
Subdural hematoma	6 (16.2%)	31 (83.8%)	1.22 (0.63–2.45)	0.13
Subarachnoid hemorrhage	5 (14.3%)	31 (85.7%)	0.88 (0.30–1.15)	0.18
Brain contusion	16 (39%)	25 (61%)	2.6 (1.07–4.09)	0.003*
**Mode of intervention given**				
Surgical	44 (42.3%)	60 (57.7%)	1.7 (1.03–3.82)	0.0001**
Non-surgical	27 (9.1%)	271 (90.9%)	1	
**Posttraumatic amnesia**				
Yes	40 (25.6%)	116 (74.4%)	1.99 (1.08–3.48)	0.005*
No	31 (12.6%)	215 (87.4%)	1	
**History of comorbidities**				
Yes	37 (50.7%)	36 (49.3%)	1.56 (1.08–3.86)	0.04*
No	34 (10.3%)	295 (89.7%)	1	
**History of alcohol abuse**				
Yes	48 (45%)	59 (55%)	3.1 (1.89–5.23)	0.000**
No	23 (7.8%)	272 (92.2%)	1	

*P-value < 0.05, **P-value < 0.001, AHR- Adjusted hazard ratio, CI-Confidence interval

## Discussion

Various studies on patients with TBI reported the incidence of early PTS within the range of 0.4 to 26.7% [7, 14, 16, 18, 20]. This study indicates that there is a high incidence of early PTS, which was observed in 17.7% of patients with moderate or severe TBI. This finding was higher than the report of previous studies conducted in the United States (1.9%) [16], Australia (2.7%) [9], Thailand (5.6%) [7], Italy (7%) [2], Shenzhen, China (2.8%) [18] and Norway (5.6%) [17], New Delhi, India(2.1%) [19], and Nigeria(10.1%) [21]. Likewise, a recent study based on a nationwide trauma database in the USA found that early seizures occurred in 0.4% of patients with TBI [14]. However, it was slightly lower than the result reported by the United States Brain Injury Research Center (26.7%) [11]. This large variation could be due to differences in the study population, definitions, and method of seizure detection (clinical observation or continuous electroencephalogram analysis).

Older age, severe TBI, EDH, brain contusion, surgical intervention, post-traumatic amnesia, and alcohol abuse were all found to be significant predictor factors of early PTS. This study found that age was a strong predictor of early PTS, which has been also documented as a risk factor for early PTS in the United States of America (USA) [14]. However a study in Victoria reported that younger age was a significant risk factor for developing early PTS [9]. In this study, alcohol abuse is a strong predictor of early PTS. This is consistent with studies from Norway [17], Southern Thailand [7], and the USA [14] which reported alcoholism as a strong risk factor for early PTS. This may be due to alcohol consumption may decrease seizure threshold by acting on gamma-aminobutyric acid receptors [17, 25]. In various literatures, moderate and severe brain injuries are independent risk factors associated with early PTS [9, 14]. The study found that the risk of developing early PTS was 2 times higher in those patients with severe TBI. This is in agreement with previous studies where severe brain injury has been identified as a potential predictive factor for the development of early PTS [7, 9, 14, 17, 19, 20]. This might be, patients with severe TBI have severe brain tissue damage which stimulates exchanges in extracellular ions and excessive release of glutamate, leading to enhanced excitatory connectivity which ultimately stimulates seizure activity. Thus, lower GCS scores showed an increased risk of seizure [26].

In the current study, EDH was also a significant predictor of early PTS which is supported by previous studies in Southern Thailand [7] and USA [14]. Patients with TBI who had brain contusions showed a higher risk of developing early PTS. This is supported by studies in Norway [17] and Nigeria [21]. Surgical intervention was among the strongest and statistically significant predictors of early PTS in our study, which is also reported in various previously published findings [9, 27, 28]. This might be due to neurosurgical procedures may inadvertently cause cerebral irritation and edema that leads to seizure occurrence. The study found that patients with a history of comorbidities had a high risk of developing early PTS. This is consistent with studies in USA [14]. Also, a history of medical problems at the time of injury raises the risk of developing PTS by 4.4 times in New Delhi, India [19].

The study has the following limitation: the lack of continuous electroencephalography (cEEG) monitoring and thus the limited ability to detect nonconvulsive seizures might led to an underestimation of early PTS in our study.

## Conclusion

Our study demonstrates that the incidence of early PTS was high following traumatic brain injury. Older Age, TBI severity, epidural hematoma, brain contusion, surgical intervention, posttraumatic amnesia, history of comorbidities, and history of alcohol abuse were strong predictors of early PTS. Since, early PTS can worsen secondary brain damage, knowing the predictors helps to provide an effective management plan for patients likely to develop early PTS and improve their outcome. Also, identifying patients at high risk of developing early PTS could allow for targeting clinical trials of antiepileptogenic therapies.

## Data Availability

The datasets generated and/or analyzed during the current study are available from the corresponding author on reasonable request.

## Abbreviations

AHR: Adjusted hazard ratio
CI: Confidence interval
EDH: Epidural Hematoma
GCS: Glasgow Coma Scale
PTS: Posttraumatic seizure
TBI: Traumatic brain injury
USA: United States of America
